# Intestinal microbiota has important effect on severity of hand foot and mouth disease in children

**DOI:** 10.1186/s12879-021-06748-7

**Published:** 2021-10-13

**Authors:** Chenguang Shen, Yi Xu, Jingkai Ji, Jinli Wei, Yujin Jiang, Yang Yang, Minghui Yang, Huaxin Huang, Rongrong Zou, Chunxiao Fang, Fansen Zeng, Fengxia Yang, Xinfa Wang, Jing Yuan, Jianmin Li, Xianfeng Wang, Huanming Yang, Sitang Gong, Hui Wang, Huimin Xia, Jinmin Ma, Yingxia Liu

**Affiliations:** 1 Graduate Collaborative Training Base of the Third People’s Hospital of Shenzhen, Hengyang Medical School, University of South China, Hunan, 421001 China; 2 School of Public Health, Southern Medical University, Guangzhou, 510515 China; 3 Guangzhou Women and Children’s Medical Center, Guangzhou Medical University, Guangzhou, 510120 China; 4 BGI-Shenzhen, Shenzhen, 518083 China; 5 Department of Engineering Science, University of Oxford, Oxford, OX3 7DQ UK; 6 James D. Watson Institute of Genome Science, Hangzhou, 310058 China; 7 China National GeneBank, BGI-Shenzhen, Shenzhen, 518120 China

**Keywords:** Hand foot and mouth disease, Intestinal microorganisms, *Bacteroides* and *Clostridium*, Predictive markers

## Abstract

**Background:**

The incidence of hand foot and mouth disease (HFMD) has increased in recent years, making it a very common childhood illness worldwide. The relationship between different enterovirus genotypes and disease severity is not clearly understood. Given that enteroviruses are transmitted through the gastrointestinal tract, we hypothesized that variation in intestinal microorganisms of the host might play a role in the prognosis of HFMD.

**Methods:**

We carried out a meta-transcriptomic-wide association study of fecal samples obtained from a cohort of children (254 patients, 227 tested positive for enterovirus, including 16 patients co-infectied with 2 kinds of enterovirus) with mild and severe HFMD and healthy controls.

**Results:**

We found there was no significant difference in the amount of each virus type between the mild and severe cases. Genes of enterovirus 71 (EV71) and coxsackievirus A (CV-A) from the severe and mild cases did not show significant clustering. *Clostridium* sp. L2-50 and *Bacteroides stercoris* ATCC 43183 were enriched in the guts of children with severe HFMD and KEGG enrichment was found between mild and severe cases.

**Conclusions:**

Intestinal microorganisms appear to interact with enterovirus to determine the progression of HFMD. Genes of *Bacteroides* and *Clostridium* may be used as predictive markers for a more efficient prognosis and intervention. The enrichment of intestinal bacteria genes with functions may facilitate the development of severe symptoms for HFMD patients.

**Supplementary Information:**

The online version contains supplementary material available at 10.1186/s12879-021-06748-7.

## Background

Hand foot and mouth disease, caused by various enteroviruses, is a common disease in children worldwide. Enterovirus 71 and coxsackievirus A16 (CV-A16) were the most common HFMD-causing pathogens reported; however, since 2010, coxsackievirus A6 (CV-A6) emerged as the most common serotype in Shenzhen, China [1, 2], and was found in an outbreak of CV-A6-associated HFMD in China since 2013 [3]. It even became a lethal strain in northeast China [4]. Besides, since 2012, coxsackievirus A10 has been the most common serotype reported in Wuhan, China [5]. Moreover, coxsackievirus A4 (CV-A4) and A10 (CV-A10) have become increasingly common in recent years, which has coincided with the emergence of more severe cases of HFMD. Although EV71 has been one of the major causative agents for severe cases of HFMD in the last decade, the relationship between different genotypes of enteroviruses and disease severity remains unclear [6–10]. Patients with severe symptoms usually develop neurological and systemic complications rapidly. In some enterovirus-positive cases, infection can be fatal within 3–5 days. However, currently, there is no useful clinical indicator for predicting the severity of disease upon diagnosis.

Although enteroviruses are the pathogens causing HFMD in children, they also belong to common intestinal microorganisms. Therefore, bacterial colonization in the intestine may be associated with emergent disease [11]. Alternatively, these microorganisms could play an important role by acting as a barrier against pathogen invasion [12, 13]. Indeed, intestinal microorganisms have been shown to cause certain types of human diseases, including type I diabetes or inflammatory bowel diseases [14], and in some cases, commensal microbes in the intestine can influence the presence of viruses such as norovirus [15], or can influence risk of plasmodium falciparum infection [16]. Specifically, intestinal bacteria can interact with viruses to alter the intestinal physiology, leading to pathology [17]. Therefore, intestinal microorganisms might also interact with enterovirus, which would influence the HFMD status.

To evaluate the potential association between the intestinal microbial community and disease severity of HFMD and identify novel predictive clinical biomarkers for severe cases, we performed total-RNA-seq shotgun sequencing on fecal samples obtained from individuals with HFMD, and compared the intestinal microorganisms between mild and severe cases and identified enrichment of specific bacteria in severe cases. Accordingly, we built an index model for predicting whether an individual will develop a severe case of HFMD based on intestinal microbiota composition. This study should enhance our understanding of HFMD beyond the current focus on enteroviruses and are expected to serve as guidance for developing new clinical treatments.

## Methods

This study was conducted at Shenzhen Third People’s Hospital and approved by the Ethics Committees of Shenzhen Third People’s Hospital. Informed consent was collected from all of our patients by their parent or legal guardian.

### Clinical patient classification

All the samples were collected from pediatric patients at Shenzhen Third People’s Hospital and Guangzhou Women and Children’s Medical Center, and were classified into 4 groups: healthy (H), mild case (M), severe case (S), and severe case after one week of treatment (A). The distinction for classifying a case as mild or severe was made according to the World Health Organization’s Diagnostic Guide of Hand Foot and Mouth Disease [18]. Specifically, a severe case should be associated with encephalitis symptoms. Physical examination results were collected as healthy samples, and healthy cohorts are defined as results with normal body temperature, without lung wet sound, without skin rash, and enteroviruses negative. All of the samples were collected before drug treatment (3–5 days after the onset of disease) or after a week treatment for the second sampling to severe cases. Finally, fecal samples collected from 100 severe cases (S), 30 after-treatment cases (A), 154 mild cases (M), and 13 healthy volunteers (H) were sequenced and compared. This study was approved by the Institutional Review Broad (BGI-IRB) in Shenzhen.

### Sample collection and RNA extraction

In total, 297 fecal samples were collected from patients with suspected HFMD who visited the Shenzhen Third People’s Hospital and Guangzhou Women and Children’s Medical Center as well as from volunteers visiting for physical examinations. The mean age of all individuals was 25.5 months (range 5–104 months); 184 were male and 113 were female. All samples were maintained at − 80 °C and shipped on dry ice before sample processing.

Total fecal RNA was extracted from the feces supernatants that were obtained from dissolving a 0.5-cm^3^ feces sample in 1 mL phosphate-buffered saline. The RNA was finally eluted with 60 μL of Nuclease-free Water using the QIAamp Viral RNA Mini Kit (Qiagen, Inc., Hilden, Germany) [19], according to the manufacturer’s instructions; the reagent dosage was adjusted to be equal to the volume of the samples. The quality, quantity, and integrity of total RNA were evaluated using an Agilent 2100 BioAnalyzer (Agilent Technologies, Santa Clara, CA, USA) and Agilent BioAnalyzer RNA Nano LabChip.

### Library preparation and sequencing

About 2 μg of total RNA was fragmented with Covaris E210. Using these short fragments as templates, random hexamer primers were used to synthesize the first-strand cDNA. The second-strand cDNA was synthesized in the reaction buffer containing dNTPs, RNase H, and DNA polymerase I. Short double-stranded cDNA fragments were purified with a QIA Quick PCR extraction kit (QIAGEN), eluted with the Elution Buffer, and then end-repairing was performed with the addition of 3′-A overhangs. Next, the short DNA fragments were ligated to Ion Torrent-compatible barcoded adapters. DNA fragments of a selected size (200 bp) were gel-purified and amplified by polymerase chain reaction (PCR). AMPure beads (Beckman Coulter) were used to purify the resulting library, and an Agilent 2100 BioAnalyzer (Agilent Technologies) and Agilent BioAnalyzer DNA High-Sensitivity LabChip (Agilent Technologies) were used to determine the concentration and size of the library [20]. The libraries were pooled in equal volumes and emulsion PCR-amplified [21] on ion sphere particles (ISPs) using the Ion One Touch instrument (Thermo Fisher Scientific, Waltham, MA, USA). The template-positive ISPs were enriched on the Ion One Touch ES instrument (Thermo Fisher Scientific) using Ion PI™ Template OT2 200 Kit v3 according to the manufacturer’s instructions. Ion PI™ Chips (Thermo Fisher Scientific) were used for sequencing on the Ion Torrent Proton platform (Thermo Fisher Scientific), which was conducted by Beijing Genomics Institute. The raw sequencing reads from the Ion Torrent Proton instrument were sorted by barcode. Each sample produced an average of 28 million reads. This study has not set an independent negative control for each sequence because the sequencing was done in a quality-controlled library, which enabled timely detection of the microorganisms of reagents and environment through a HELA cell sequencing for control.

### Identification of enterovirus type

The raw reads were mapped to the human genome (hg19) using TMAP (v. 3.4.1, -g 0 -a 1 stage1 map4), and unmapped reads were first mapped to the enterovirus reference sequences (downloaded from the National Center of Biotechnology Information [NCBI], manually curated, including 3901 sequences, last updated: May, 2014) through TMAP (v. 3.4.1, -g 0 -a 1 stage1 map4) and then filtered (minimum read length: 50; coverage map of read: 80%). Reads mapped to the enterovirus reference sequences were clustered, and then highly similar sequences were removed using CD-HIT [22] (-c 0.99). The raw reads were first assembled into contigs using IDBA-trans [23] (v. 1.1.1, -mink 15; -seed_kmer 25; -min_contig 50; -no_local) followed by Phrap [24] (v. 1.080812, -minmatch: 10; -maxmatch: 100; -minscore: 30; -vector_bound: 3; -maxgap: 5). To determine the enterovirus type, the contig sequences were compared to the manually curated enterovirus reference database for the VP1 region using BLAST (-W 28 -a 10-e 0.001 -b 5 -m 8 -F F) and TMAP (-g 0 -a 1 stage1 map4), respectively. A nucleotide sequence homology of at least 75% was required for assignment to the same genotype. In our working scheme, nucleotide sequences that respectively mapped to the same enterovirus type using BLAST and TMAP were further validated with the Enterovirus Genotyping Tool (v.0.1) [25].

### Quantification and normalization of meta-transcriptomic expression

In order to estimate the whole transcriptome of the gut microbiome, the gut gene set containing 9,879,896 genes was selected as reference [26]. The samples with reads number less than 90% unmapping to human genome were discarded. Subsequently, and all of the reads were mapped to the reference genes (TMAP, minimum read length: 50; coverage map of read: 80%). The genes should at least be mapped 2 unique reads at different location. The mapping result were filtered as described before. Then, the gene expression level was calculated as reads per kilobase per million mapped reads (RPKM):

$${\text{RPKM}} = 10^{9} *{\text{C}}/{\text{NL}}$$

In which *C* represents the number of reads uniquely mapped to a given gene, *N* is the number of reads uniquely mapped to all genes, and *L* indicates the total length of the given gene. For genes with more than one alternative transcript, the longest transcript was used to calculate the RPKM, which was then directly used to compare the differences in gene expression among samples.

### Rarefaction curve for the intestinal genes and samples filter

For each sample, all raw sequenced reads were cut at every one million reads to evaluate whether the gut microorganism genes were sufficiently sequenced to allow for differential analysis between the patient groups. Rarefaction for the intestinal microbial gene content of all samples was used to evaluate the gene saturation level of the samples. The number of genes in each group (from one sample to all samples) was calculated after 100 random samplings with replacement.

### Gene marker-based classification

In order to identify microorganism genes that could be used as potential markers for distinguishing between severe and mild cases of HFMD, all of the differentially expressed genes (DEGs) between any two groups were determined using the Wilcoxon rank-sum test (p < 0.01). The minimum redundancy–maximum relevance (mRMR) feature selection method described by Peng [27] was used to calculate the redundancy coefficient for each gene, which was used to sort the genes. The accuracy of each model was evaluated by leave-one-out cross-validation (LOOCV) to find the optimum subset for building a linear discrimination classifier. We chose the lowest error rate model as the final model to predict the remaining samples as a severe or mild case. The linear regression formula was as follows:

$${\text{F}}\left( {\text{x}} \right) = {\text{a}}_{0 } + {\text{a}}_{1} {\text{x}}_{1} + {\text{a}}_{2} {\text{x}}_{2} + \ldots + {\text{ a}}_{n} {\text{x}}_{n}$$

where “*X*” refers to the expression of the genes selected from mRMR selection and *a* indicates the redundancy coefficient of each gene.

### Meta-transcriptomic-wide association study between groups

A meta-transcript linkage analysis modified from a metagenomic linkage group (MLG) analysis [28] was carried out to evaluate the abundance of the microbiome. In order to estimate the best parameter, we selected 608,897 genes from 50 microbial species that belonged to mild and severe samples, which were subjected to different tests: (a) gene coverage (0, 50, 70, 80, 90, 95%), which indicates the percentage length mapped by reads; (b) sample number cutoff (0, 1, 2, 3, 4, 5, 6, 7, 8, 9, 10%), which indicates the number of genes detected in a given number of samples; and (c) the minimum number of MLG size. All species were identified with an annotation accuracy of 97.1% for parameter coverage = 0, sample cutoff = 7%, and MLG size > 11/219,531 (Additional file 1: Fig. S1). All of the comparisons of MLG abundance between the different groups were based on these parameters.

### Functional analysis of the microbiome between groups

A Wilcoxon rank-sum test was used to identify potential genetic markers distinguishing the different groups according to HFMD severity. The candidate genes were annotated to the Integrated Reference Genome of the Human Gut Microbiome (IGC) Kyoto Encyclopedia of Genes and Genomes (KEGG) database [26]. The percentages of gene markers belonging to each KEGG category (KEGG class level 2) out of the total of group-1-enriched or group-2-enriched gene markers were designated as the comparison parameter. Fisher’s exact test was used to calculate the significance level in the functions.

### Hypothesis testing

In our multiple hypothesis test, we used the q-value to measure the false-positive rate (false discovery rate, FDR) [29]. Based on previous definitions, we define Q as the proportion of false discoveries among the discoveries $Q = \frac{V}{R}$. The FDR is given by [30]:

$$FDR = Q_{e} = {\text{E}}\left[ Q \right] = {\text{E}}\left[ {\frac{V}{V + S}} \right] = {\text{E}}\left[ \frac{V}{R} \right]$$

where $\frac{V}{R}$ is defined as 0 when $R = 0$. This value should be below a threshold α (or *q*).

### Viromics analysis

All of the virus, bacteria, and fungi complete genome sequences were collected from the NCBI ftp site, and all sequences were merged for constructing a microbiome database using Kraken [31]; in addition, all of the clean reads of samples were classified using Kraken. The relative abundance of each species was calculated according to the RPKM value described above, where *C* is the number of reads uniquely classified to microbe species, *N* is the number of reads uniquely classified to all microbe species, and *L* is the genome length of the classified species. A Wilcoxon rank-sum test was used to identify whether viruses other than enterovirus differed or showed enrichment between mild and severe cases. In order to confirm the right result, all the clean Reads belonging to virus (including herpesviruses) were checked through an independent alignment by BLAST to NT database. The power analysis was done used software G*Power3.1.9.7, the result sample size should more than 73 with the actual power 0.85.

## Results

### Identification of enterovirus genotypes in patients with HFMD by next-generation sequencing (NGS)

**Fig. 1:**
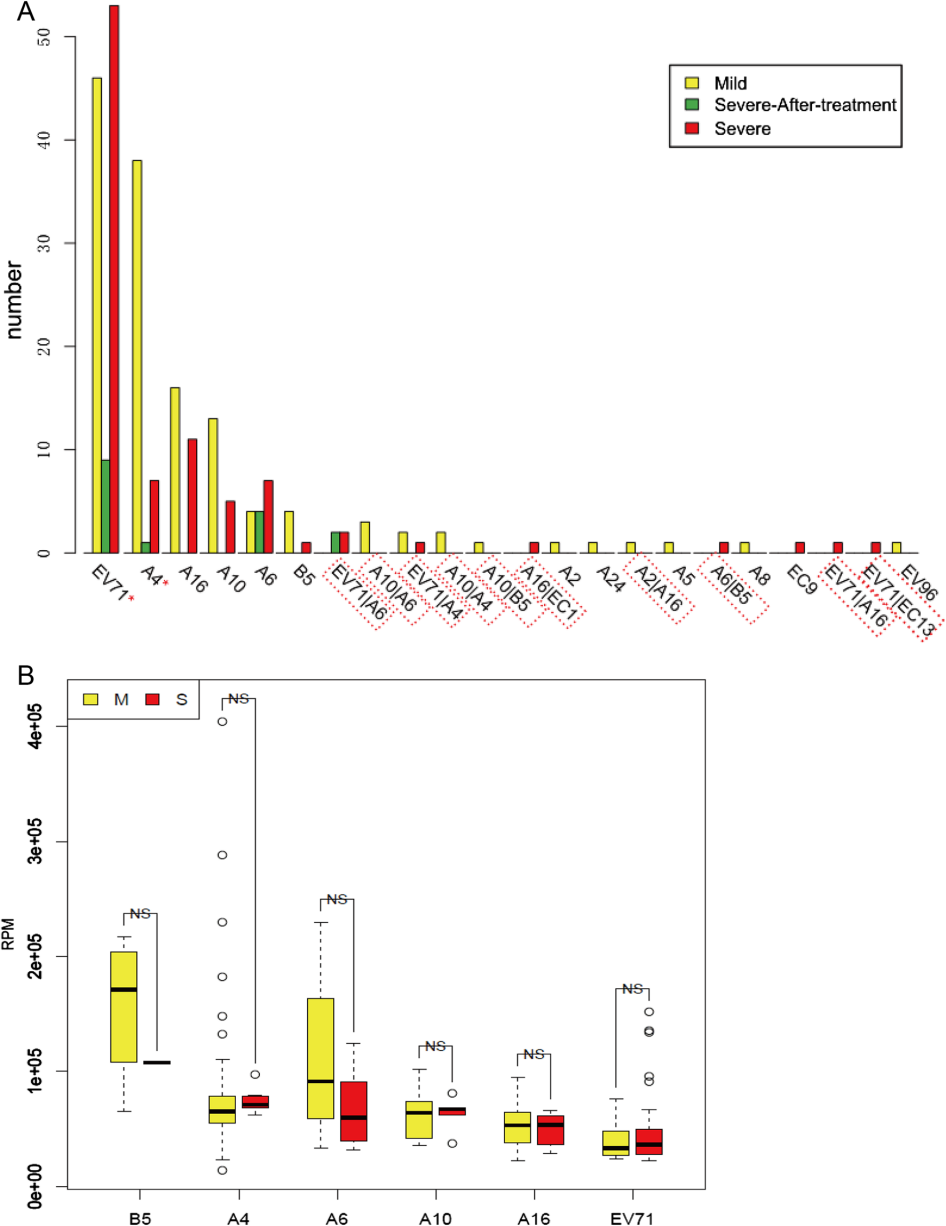
Enterovirus type identification in HFMDs individual by NGS. **A** Individuals distribution of the samples with enterovirus type. Red box highlight the co-infection samples. Asterisk means statistical significance difference between mild and severe cases number for enterovirus (Fisher’s exact test p < 0.05). EV71: Human Enterovirus 71; start with “A”: Coxsackievirus A. start with “B”: Coxsackievirus B; start with “EC”: ECHO-virus. **B** Main virus amount by NGS compare between severe and mild cases. RPM: reads number per million sequenced reads. *NS* not significant

Of the 254 patients (group M and S), 227 (89.4%) were tested positive for enterovirus, including 16 (6.3%) patient co-infection with 2 kinds enterovirus. Enterovirus positive was detected from about 92.0% (92/100) of the severe cases and 53.3% (16/30) even after treatment. The co-infection rate was 6.3% (16/254) overall, and was 7% and 5.8% in severe and mild cases, respectively, with no significant difference (Fisher’s exact test, p > 0.05). And 14 different virus genotypes were identified with different detection rates: EV71 (41.7%, 106/254), CV-A4 (19.7%, 50/254), CV-A16 (11.8%, 30/254), CV-A10 (9.4%, 24/254), CV-A6 (6.7%, 17/254), CV-B5 (2.8%, 7/254), CV-A2 (0.8%, 2/254), CV-A5 (0.4%, 1/254), CV-A8 (0.4%, 1/254), CV-A24 (0.4%, 1/254), HEV9 (0.4%, 1/254), HEV13 (0.4%, 1/254), HEV1 (0.4%, 1/254), and EV96 (0.4%, 1/254). EV71 infection was predominant among the HFMD cases (Fig. 1A). EV71 was detected at a higher rate in the severe cases, whereas CV-A4 was detected at a higher rate among the mild cases (Fisher’s exact test, p < 0.05). However, there was no significant difference in the amount of each virus type (calculated as RPM) between the mild and severe cases (Fig. 1B). NGS could identify a greater number of co-infections in addition to EV71 plus CAV16 infection when compared to quantitative real-time polymerase chain reaction (RT-PCR); overall, 10 kinds of co-infection were identified in this study, and 4 virus types showed co-infection with EV71. Some cases were identified to be enterovirus-negative, indicating that the enterovirus load was low or perhaps a true negative, with a rate of 8% (8/100) and 12.3% (19/154) in severe and mild cases, respectively, with no significant difference between severe and mild case (Fisher's exact test, p > 0.05).

### Phylogenetic analysis of the main enterovirus types

**Fig. 2:**
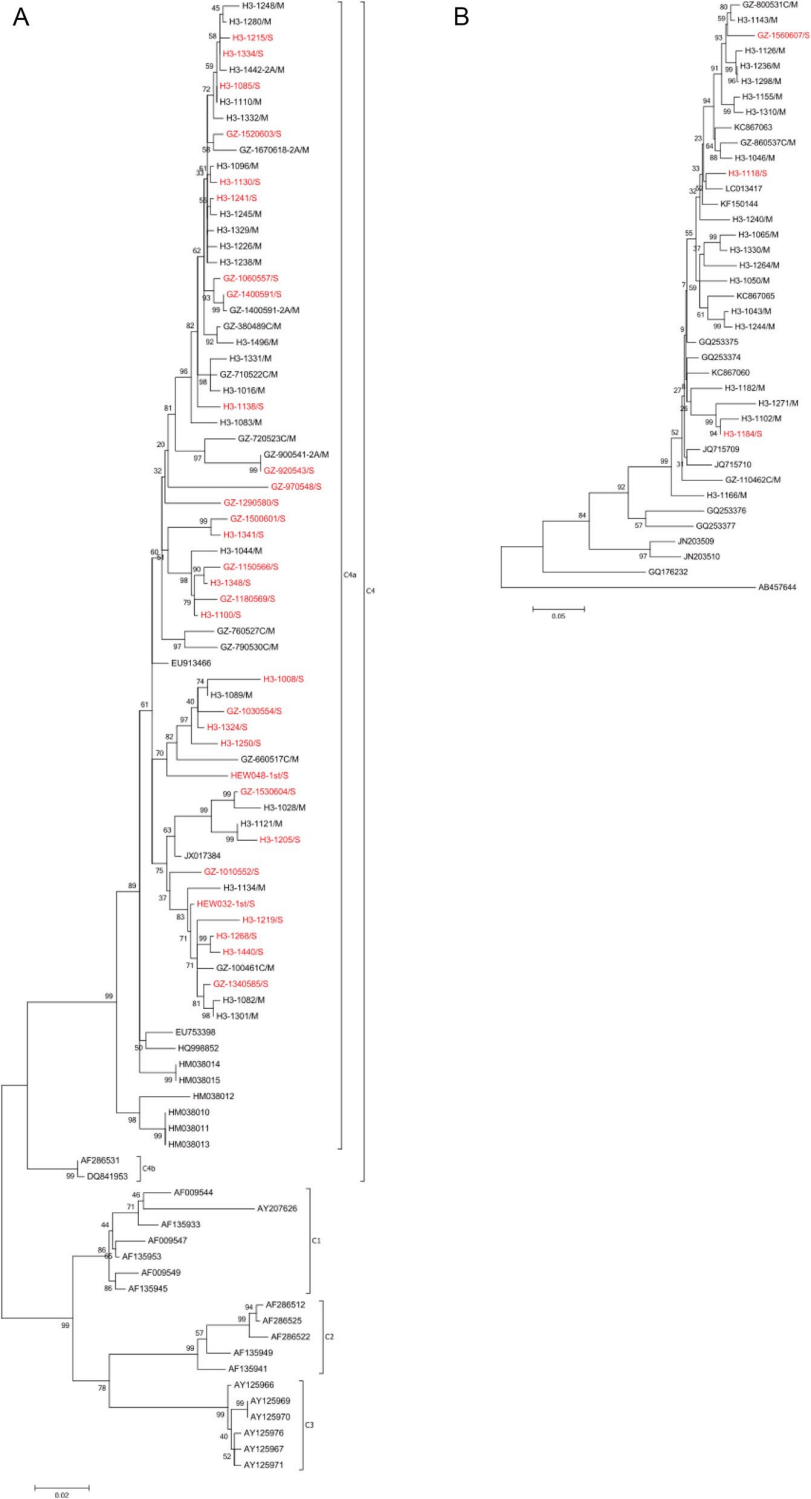
Enterovirus phylogenetic tree between mild and severe case (VP1). **A** The phylogenetic tree of the enterovirus 71. **B** The phylogenetic tree of the coxsackievirus A4. Red color highlighted in branch means severe cases and black means mild case. The branch name started with “H3” or “GZ” was the sequences obtained in this study. The phylogenetic tree was constructed by software MEGA 6 with Maximum Likelihood method

All of the samples that were EV71- and CV-A4-positive were selected for phylogenetic analysis, since these were the most frequent types of infections in the study samples. The genomes of EV71 and CV-A4 strains were assembled, and the whole VP1 gene sequences were identified via the alignment to the reference sequence and applied to phylogenetic analysis with MEGA 6 [32]. All of the EV71 VP1 genes from the study samples belonged to the C4 genotype and C4a sub-genotype (Fig. 2A), similar to previous findings [5]. Although EV71 caused more severe cases than the other enteroviruses did, the severe and mild cases did not cluster in a clear pattern according to the phylogenetic tree, neither did CA-A4 (Fig. 2B); instead, all of the severe and mild cases were basically in the same branches. In addition, the sequences covering more than 80% of the genome were selected for phylogenetic analysis of EV71 (Additional file 1: Fig. S2A) and CV-A4 (Additional file 1: Fig. S2B) to identify any genome-wide differences. All of the EV71 genomes were classified into 2 clusters: one cluster corresponding to a strain isolated from Guangzhou in 2009, and the other corresponding to a strain isolated from Canada in 2006 to 2007. However, the severe and mild cases did not show significant clustering, which was also the case for the CV-A4-positive samples. These results implied that other factors may influence the severity of HFMD caused by the same type of enterovirus.

### Contribution of viruses other than enterovirus to HFMD

Seven patients that were diagnosed with a severe case of HFMD were enterovirus-negative according to NGS identification. This indicates that the enterovirus load was either very low or was in fact negative. We selected another 7 samples with the highest enterovirus load to evaluate whether there was any other virus difference between the groups. We did not identify any significant difference in other viruses besides enterovirus between the low enterovirus-load group and the high enterovirus-load group (Fisher’s exact test, p > 0.05).

We further evaluated all of the differences in viruses between mild and severe cases to evaluate the contribution of virus type to disease severity. We found that several typical plant viruses differed between mild and severe cases (Table 1). These plant viruses may be food-borne; however, there is little information available about their potential influence on children’s health. Nevertheless, these plant viruses were detected at different rates in fecal samples from mild and severe cases, indicating difference that may exist in the virus clearing abilities between the groups.

**Table 1:** Difference of virus between mild and severe cases (besides enterovirus)

Species	Mild sample (114)	Severe sample (74)	All (188)	Fisher test	Wilcoxon	Type
Groundnut ringspot and Tomato chlorotic spot virus reassortant	2	12	14	0.0003	0.0002	M < S
Cucumber green mottle mosaic virus	13	22	35	0.0021	0.0023	M < S
Porcine endogenous retrovirus E	9	18	27	0.0025	0.0009	M < S
Magnaporthe oryzae chrysovirus 1	1	8	9	0.0027	0.0019	M < S
Watermelon mosaic virus	0	6	6	0.0033	0.0021	M < S
Penicillium chrysogenum virus	8	17	25	0.0035	0.0016	M < S
Cynomolgus macaque cytomegalovirus strain Ottawa	6	14	20	0.0063	0.0032	M < S
Ictalurid herpesvirus 1	6	14	20	0.0063	0.0021	M < S
Geobacillus virus E2	0	5	5	0.0087	0.0051	M < S
Tomato spotted wilt virus	2	8	10	0.015	0.0068	M < S
Canarypox virus	0	4	4	0.0228	0.0125	M < S
Human herpesvirus 5	14	2	16	0.0298	0.0188	M > S
Oryctes rhinoceros nudivirus	2	7	9	0.0299	0.014	M < S

Mild sample (114): positive samples number in mild cases, total 114. Severe sample (74): positive sample number in severe cases, total 74. All (188): all positive sample number, total 188. Fisher test: Fisher’s exact test, p-value. Wilcoxon: wilcoxon rank-sum test, p-value. Type: the amount enriched in Mild (M > S) or Severe (M < S)

### Sample saturation of raw sequence data and gut microorganism genes

**Fig. 3:**
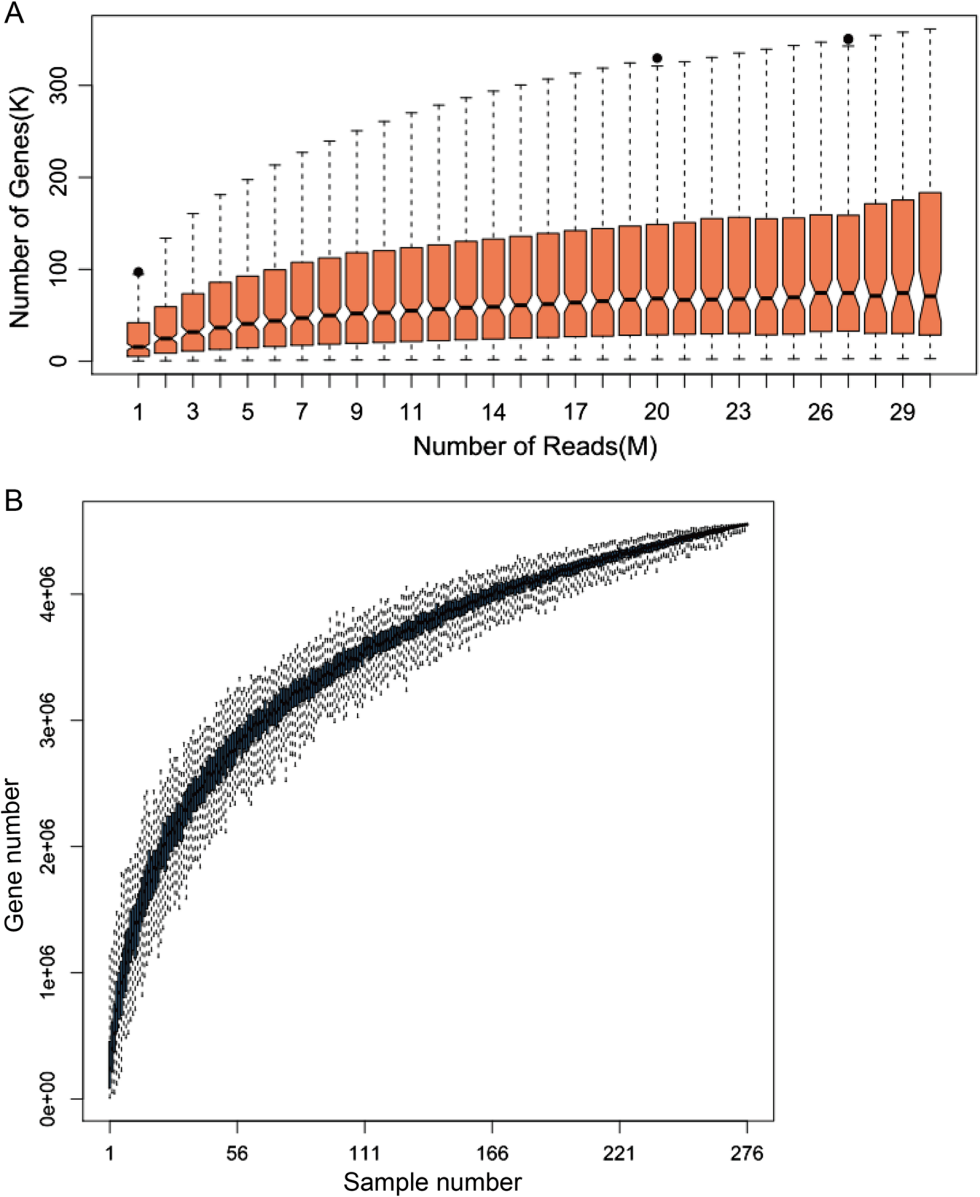
Sample saturation of all sample for gut meta microbe genes. **A** cumulative sequence data for each sample. **B** Accumulated samples for all the genes. Each sample number was randomly repeated for 100 times

For each sample, a sufficient number of sequences should be sequenced to prepare for effective comparison among groups (Fig. 3A). The number of new genes identified increased gradually after the reads reached 9 million. Overall, all samples sequenced more than 20 million reads, which was considered to be suitable for statistical and association analyses. Figure 3B shows that the gene number increased rapidly when the sample number was below 50, but increased slowly when the sample number was more than 100. Although the gene number continued to increase even when the sample number reached 276, few new genes were detected at this point. Finally, more than 4.5 million genes were detected in the total dataset of 9.9 million genes.

### Microorganism gene marker index and predictive model for distinguishing between severe and mild cases

**Fig. 4:**
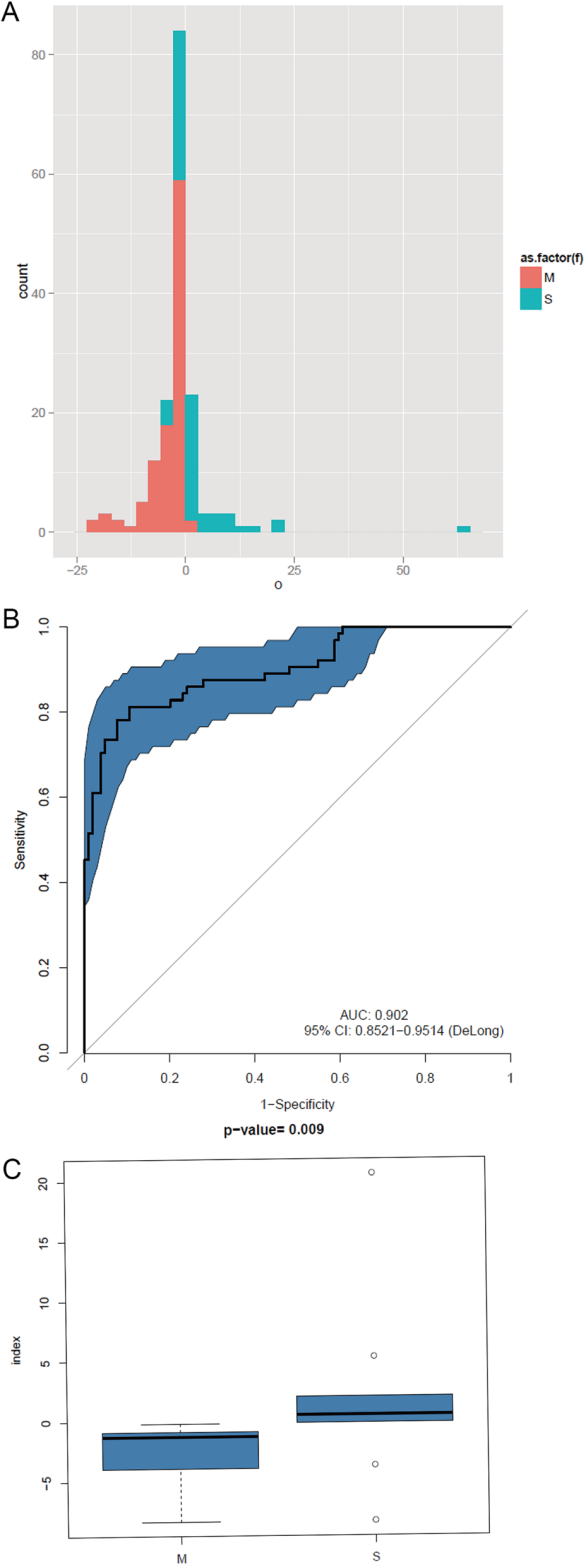
Severe and mild case index model of Enterovirus positive samples. All 20 genes at lowest error rate: 20.83%. **A** For each individual, a index was calculated to evaluate the risk of severe HFMD disease (Training: M:S = 104:64). The histogram shows the distribution of indices for all individuals. Red means Mild case and blue means severe case. **B** Receiver operating characteristic curve of the index model. The area under the ROC curve (AUC) of classifier is 0.9. **C** new samples for test using the index model (Verify: M:S = 10:10). Mild case and severe case were distinguished by the index model (p < 0.01)

We selected 104 mild cases and 64 severe cases randomly that were enterovirus-positive to search for marker genes to distinguish between mild or severe cases. Overall, we identified 52,290 DEGs, and the proportion of DEGs was higher in the younger cases (Additional file 1: Fig. S3); this may explain the higher severity risk in younger cases. The contribution coefficient of all of the identified DEGs was calculated using mRMR. Ultimately, the linear combination of 20 genes with the lowest error rate was used to distinguish between mild and severe case (Additional file 1: Fig. S4). These genes were then used to build a linear model and calculate the index value. In this model, these 20 genes distinguished between mild and severe cases at the lowest error rate of 20.83% (Fig. 4A and B). The model’s discriminatory ability was evaluated with the receiver operating characteristic curve (ROC), and the area under the ROC curve (AUC) of the classifier was 0.9 (Fig. 4B). To verify the model prediction accuracy, the index values were calculated for an additional 10 mild case samples and 10 severe case samples, and the Wilcoxon rank-sum test showed that the predicted result was significant (p < 0.01) (Fig. 4C). Of all 20 genes, only 7 were annotated as *Firmicutes*, *Bacteroides*, *Prevotella*, *Desulfovibrio*, *Mitsuokella*, and *Blautia*, whereas more than half of these genes were unknown (Additional file 1: Fig. S5).

We also selected random samples that were only EV71-positive (mild case: severe case = 31:35) in order to build a similar model to distinguish between mild and severe cases (Additional file 1: Fig. S6A and B). Furthermore, an additional 10 mild and severe samples were respectively predicted using the model, which was determined to be significant by the Wilcoxon rank-sum test (p < 0.05, Additional file 1: Fig. S6C). The error rate of the first model including all enterovirus-positive samples was higher than that of the second model including only EV71-positive samples. Furthermore, the first model and second model did not share many gene markers: only 2 markers were shared with a weight value of 0.024 and 0.021 in the first model and of 0.163 (the maximum weight value) and 0.071 in the second model (Additional file 1: Fig. S6D). Nevertheless, the weights of these 2 markers were in the same order in the two models.

### Differences in microbial species according to case severity and enterovirus type

**Fig. 5:**
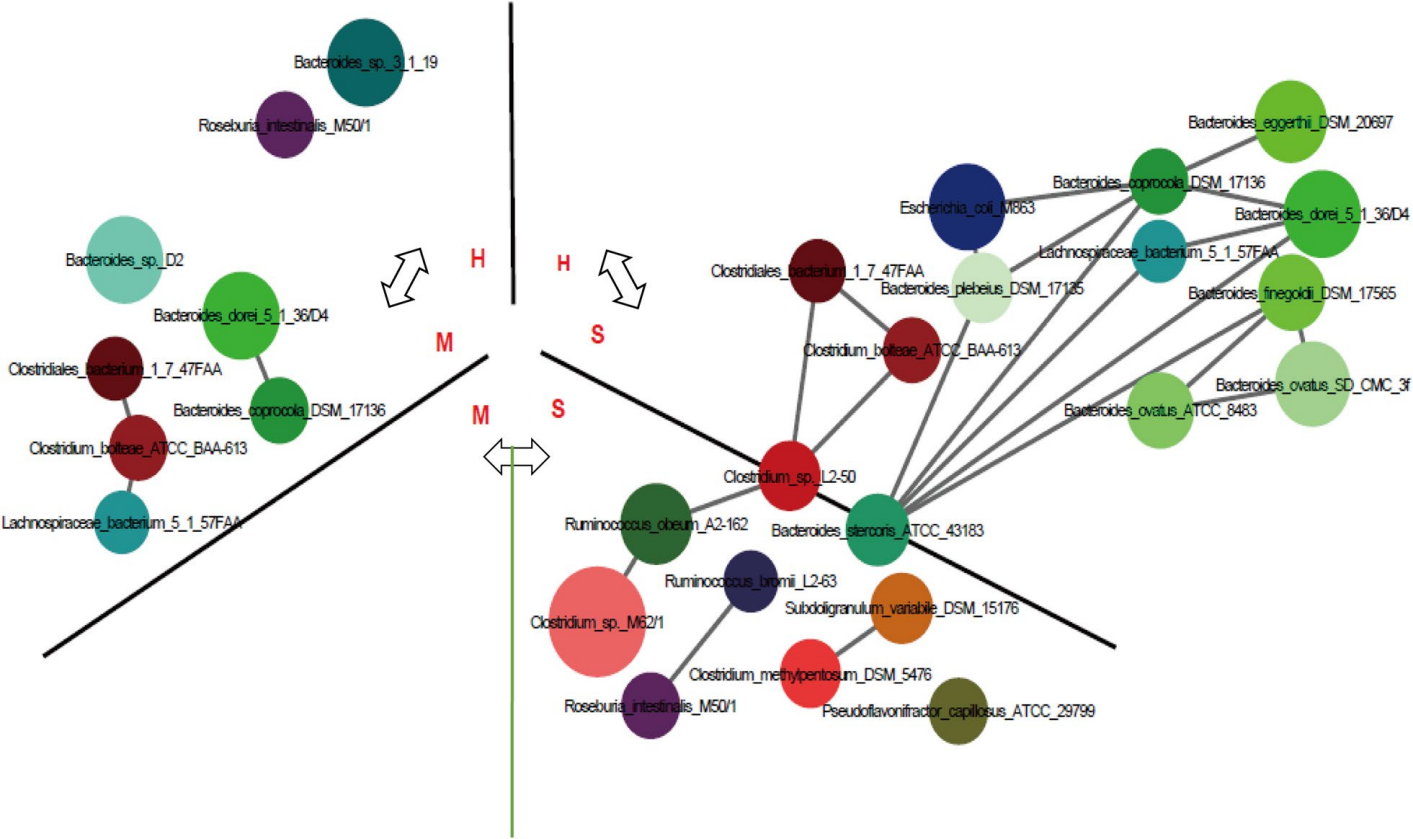
The species enrichment in H, M and S groups of age less than 24 months. White arrow means the group compare. Different circle means different MLG species. The size of the circle indicates abundance of the MLG species. The color of the circle indicates their taxonomic assignment. Connecting lines represent Spearman correlation coefficient values above 0.6 (grey) and below − 0.6 (blue)

In our data, we found that the diversity of gut microorganisms increased with increasing age until about 30 months old. Accordingly, we selected samples from subjects in the 3 groups (H, M, S) that were all less than 24 months of age to evaluate the microorganism enrichment tendency according to disease status, irrespective of age effects (Fig. 5). For the comparison between the H and M groups, *Bacteroides* sp. 3_1_19 and *Roseburia intestinalis* M50/1 were enriched in the H group, whereas *Bacteroides, Clostridiales, Clostridium,* and *Lachnospiraceae* were enriched in the M group. Comparison between the H and S groups showed enrichment of *Clostridiales, Clostridium, Bacteroides, Escherichia*, and *Lachnospiraceae* in the S group. Five of 6 bacteria were shared between M vs H group and S vs H group, enriched in M and S group respectively, but more bacteria enriched in S. Furthermore, in the comparison between the M group and S group, *Ruminococcus, Clostridium, Roseburia, Bacteroides,* and *Pseudoflavonifractor* were all enriched in the S group*.* Enrichment of *Clostridium* sp. L2-50 and *Bacteroides stercoris* ATCC 43183 were common in the comparisons of the M vs. S and H vs. S groups.

**Fig. 6:**
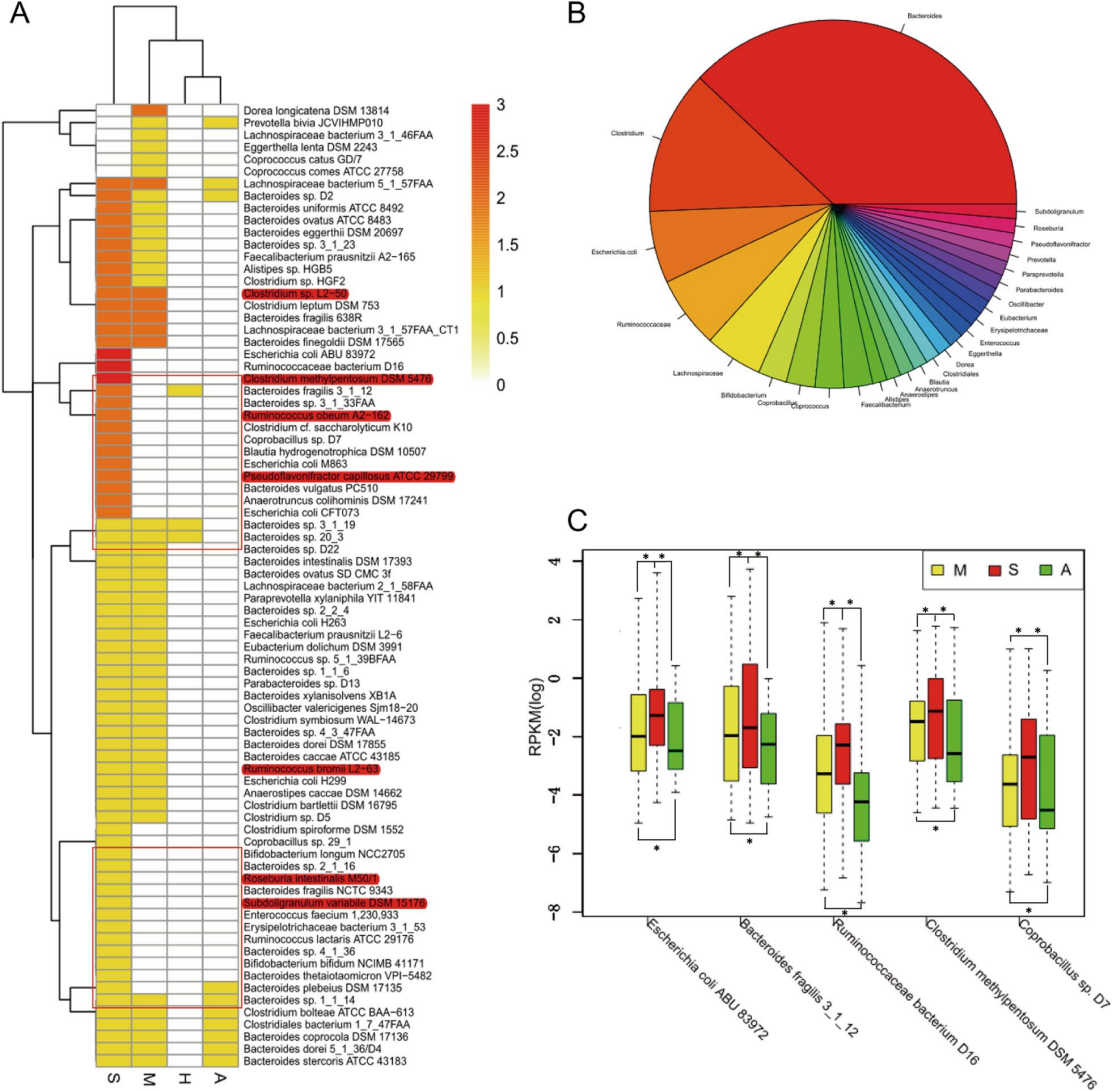
MLG species enrichment in 4 groups. **A** The cluster between species and groups. Each species was compared between groups, one enriched was marker 1, and the most enriched was 3, the range is from 0 to 3. The highlighted text in red represents the microbes which enriched in the server group, and the box section represents the clusters which these microbes belong to. **B** The pie chart of the genus of MLG species. **C** The 5 bacterias which different in “M < S > A” in all samples (wilcoxon rank-sum test,*: p < 0.05). Group marker: A: after treatment; H: health; M: mild; S: severe

Enrichment comparisons were also carried out for all samples to evaluate the overall variation in the microorganisms among the groups. We selected all species showing a significant difference between any two groups (based on the Wilcoxon rank-sum test, p < 0.01) and conducted a cluster analysis between the groups and species (Fig. 6A). The H and A groups were clustered together in one branch, whereas the S and M groups were on two independent branches. Most of the bacteria were enriched in the S group followed by the M group. The most common bacteria species were from *Bacteroides* (37.97%) followed by *Clostridium* (12.66%), as described above (Fig. 6B). The comparison between the M group and S group showed that 10 species of bacteria were unidirectionally enriched in the S group. They belonged to *Bacteroides, Clostridium, Ruminococcaceae, Eubacterium*, and *Escherichia,* and some species were positively correlated.

There were 5 kind of bacteria that showed a significant difference between the M and S group enriched in S, the S and A group enriched in S, and the A group and M group enriched in M (“M < S > A < M”; Wilcoxon rank-sum test, p < 0.01) in all samples (Fig. 6C): *Escherichia coli* ABU 83972, *Bacteroides fragilis* 3_1_12, *Ruminococcaceae bacterium* D16, *Clostridium methylpentosum* DSM 5476, and *Coprobacillus* sp. D7. The highest abundance of these bacteria was found in the S group, followed by the M group, with the lowest abundance in the A group. This suggests that a higher abundance of these bacteria may contribute to increasing the disease severity, and that the abundance would decline after treatment.

There were 4 types of coxsackievirus A (CV-A) detected (A4, A16, A10, A6) in more than 30% of all samples of both severe and mild cases. However, there were few differences in the microorganisms between these CV-A-positive groups. Therefore, all of the CV-A-positive samples were pooled as one group and compared to the EV71-positive samples as another group, and similar enrichment analysis was conducted. Both the gene and species diversity were significantly different between the CV-A group and EV71 group (sample number CV:EV71 = 102:97, Wilcoxon rank-sum test, p < 0.05). The diversity of the EV71 group was slightly higher than that of the CV-A group (Additional file 1: Fig. S7A, B), and more than 10 bacteria species were unidirectionally enriched in the EV71 group, including *Bacteroides, Blautia, Ruminococcus, Clostridium, Faecalibacterium, Alistipes,* and *Pseudoflavonifractor* (Additional file 1: Fig. S7C), *Bacteroides* constituted the greatest proportion of all bacteria. Given that the proportion of severe cases was higher in the EV71-positive group than in the CV-A-positive group, and *Bacteroides* and *Clostridium* were also enriched in severe cases, these results suggest that enrichment of these bacteria could contribute to the disease severity.

### KEGG enrichment between mild and severe cases

**Fig. 7:**
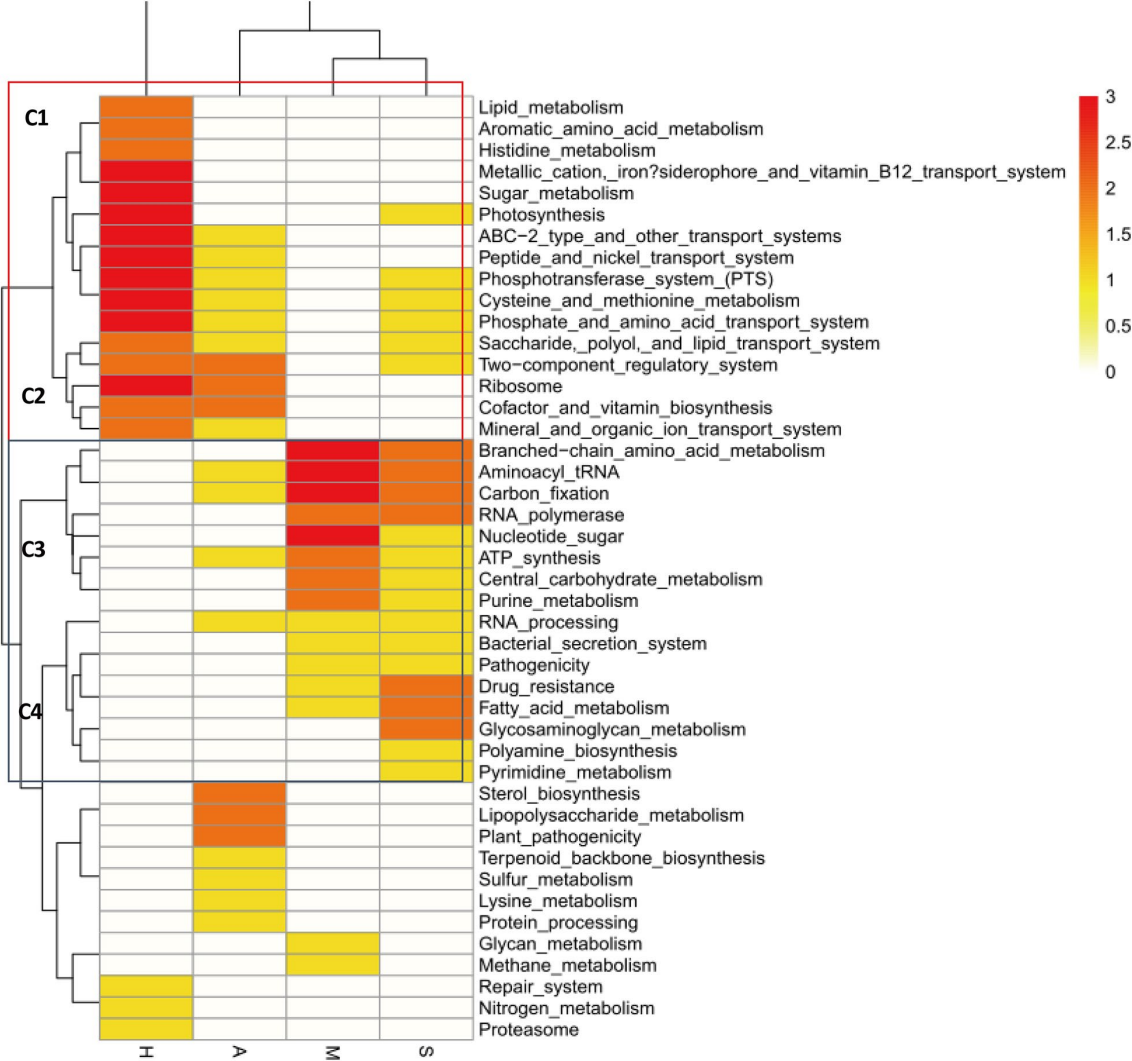
Heatmap of KEGG function difference between groups. Each function was compared between groups, one enriched was marker 1, and the most enriched was 3, the range is from 0 to 3. Cluster C1 and Cluster C2 were inclined Group H and A Cluster C3 and Cluster C4 were inclined Group M and S. Such as bacterial secretion system and Pathogenicity were enriched in HFMD case

To evaluate the function of the genes enriched between the different groups, we identified the level-2 KEGG genes, the results showed a significant difference between any two groups (Wilcoxon rank-sum test, p < 0.01) and conducted a cluster analysis between the groups and the functions (Fig. 7). The M and S groups were clustered into one branch, while group A and group H clustered in independent branches. All of the functions were classified into several clusters. Cluster C1 and Cluster C2 were associated with groups H and A, whereas Cluster C3 and Cluster C4 were associated with groups M and S. Genes associated with functions such as photosynthesis, transport systems, phosphotransferase system, cysteine and methionine metabolism, ribosome, and two-component regulatory system were enriched in the H and A groups containing healthy or treated individuals. Genes with functions related to the bacterial secretion system, pathogenicity, carbon fixation, and drug resistance were enriched in groups M and S, which included only HFMD cases. These results suggest that some metabolism enrichment may be more likely to cause HFMD.

## Discussion

### NGS and clinical identification of enterovirus types related to HFMD

The positive identification of enterovirus serves as an important indicator for a clinical diagnosis of HFMD. Most clinical diagnoses are based on one of three types of detection by RT-PCR: EV71-positive, CV-A16-positive, and universal enterovirus-positive. Therefore, RT-PCR is only capable of detecting one type of co-infection: EV71 plus CV-A16. However, EV71 may co-infect with many other enteroviruses, especially coxsackievirus, as detected in the present sample (Fig. 1A). There is currently no evidence as to whether co-infection with EV71 will increase the severity of disease. In our study, 5 of 7 cases (71.4%) showing co-infection of EV71 with another enterovirus were severe, while 53 of 99 (55.6%) cases that were only infected with EV71 were severe. Other types of enterovirus co-infection seem to increase the severe risk; however, further investigations are required to test this hypothesis since the number of cases of co-infection with EV71 in this study was small. Therefore, NGS can identify more types of co-infection quickly and may be used to provide more evidence of the association between EV71 infection and disease severity. Methods for the rapid and sensitive molecular detection of enterovirus are of paramount importance for managing HFMD outbreaks [33]. It is expected that increasing the number of samples analyzed with NGS in the future will provided new insights into HFMD.

There were 27 specimens for which enterovirus could not be detected. The samples in this study were collected 3–5 days after the onset of disease, and the samples were preserved into -80℃ refrigerator immediately until the identification by NGS to ensure the sample quality. These results indicated that the enterovirus load of these specimens was low or perhaps a true negative. In a previous study, Ho et al. investigated the ecological dynamics of the oral microbiome changes during the HFMD infection. All known vertebrate viruses in the specimens were under targeted enrichment before the detection of the viruses, and nine discriminative viruses were detected [34]. Although in Ho’s study, the specimens were from mouths, and in our study, the specimens were from feces, and the viral enrichment can improve the sensitivity of virus detection. The amount of non-infection-causing viruses presented in the samples turned out to be much lower compared to bacteria and host genetic materials, indicating that the virome of the samples in this study could be underestimated since there was no attempt in the enrichment for the viruses. Furthermore, the phylogenetic analysis and viral load test suggested that neither the genotype of enterovirus nor the amount of virus could be conclusively associated with the increased risk of severe disease. These results imply that HFMD is not only caused by enterovirus, and that other microorganisms in the intestine may also be responsible for the susceptibility and progression of the disease. Moreover, other host factors such as the immune system, genetic effects, and nutritional and hygiene status may affect the severity of HFMD.

### Intestinal microorganisms influence HFMD

In humans, bacterial cells are 10 times more abundant in the gut than in the somatic cells [35], and therefore affect the host’s health in different ways. Interest in intestinal microbiota functions has grown recently, including those related to metabolism [36], the immune system [15], and even the central nervous system [37, 38]. Koji Atarashi [39] has reported that indigenous *Clostridium* species regulate the amount and function of regulatory T cells in the colon [39, 40] and further influence the immune status of the gut. Therefore, the abundance of *Clostridium* might play a role in enterovirus infection in the gut and determine the outcomes of HFMD. EV71 is a neurotropic virus. However, to our knowledge, there are few reports of EV71-positive cases in the cerebrospinal fluid. In the present study, *Bacteroides* and *Clostridium* were enriched in the M and S groups, with particular enrichment of *Bacteroides* and *Clostridium* in the S group. Therefore, individuals with HFMD have increased colonization of *Bacteroides* and *Clostridium*, with greater abundance in more severe cases. An epidemiological study also found that very few or no mild case developed into severe cases, whereas severe cases usually progressed quickly, resulting in death in some instances within 1–3 days after the appearance of symptoms [41]. This suggests that mild and severe cases of HFMD might be independent and have different causes and mechanisms. Indeed, a previous study showed that increased colonization of *Clostridium* and *Bacteroides* in infants was associated with risks of certain diseases such as allergy or obesity [11]. One possibility is that *Clostridium* enrichment and an intensive bacterial secretion system could increase the blood–brain barrier permeability, which would induce encephalitis or neurogenic pulmonary edema, typical symptoms of severe cases.

### Vaccination and antibiotic treatment

Although some vaccines targeting HFMD have been tested [42–45], all of the vaccines developed to date are based on the EV71 type. Therefore, these vaccines would have little effect on preventing cases caused by other enteroviruses or by co-infection. Moreover, coxsackievirus appears to be causing more and more cases, including more severe cases. If the vaccine works well, the incidence of cases caused by EV71 will decrease, but potentially at the expense of an increasing number of cases caused by other enterovirus infections. In addition to the enrichment of *Bacteroides* and *Clostridium* in severe cases, the development of intestinal microorganisms tends to differ for younger children [46], which may influence their ability to resist HFMD or the severity of this disease. Therefore, the use of antibiotics to restrain bacteria enriched in severe cases may help patient recovery or the control of these bacteria, which would in turn decrease the risk for HFMD. Some intestinal probiotics agents use may reduce the *Clostridium* and *Bacteroides* in severe case.

## Conclusions

HFMD is caused by many enteroviruses, and infections have potential to develop into a severe case. Although EV71 causes the most of the severe cases, the abundance of enterovirus was not significantly different between severe and mild cases in this study. Furthermore, the enterovirus genotype was not clearly associated with disease severity. Our results suggest that the development of severe symptoms in some cases may not only depend on the enterovirus but also on enrichment of *Bacteroides* or *Clostridium* in the intestine. Moreover, our results suggest that different enteroviruses may be accompanied by different gut microorganisms; therefore, it is possible that different viruses that cause HFMD would require a different model for accurate prediction of disease severity when using the intestinal microbiome as a marker for disease prognosis. The enrichment of intestinal bacteria genes with functions such as the bacterial secretion system, pathogenicity, carbon fixation, and drug resistance may also facilitate the development of severe symptoms for HFMD patients. These results should provide useful guidance for clinical treatment.

## Supplementary Information

**Additional file 1: Fig. S1.** Best parameter select for Meta-transcript linkage group cluster. Red represents all 50 species identified; gray represents more than 30, less than or equal to 49; less than 30 is not shown. **Fig. S2.** Enterovirus phylogenetic tree between mild & severe case using whole genome wide. A: the phylogenetic tree of the enterovirus 71. B: the phylogenetic tree of the coxsackievirus A4. Red color highlight in branch means mild case and black means severe case. The branch name started with “gi” as the reference sequences. **Fig. S3.** Proportion of DEGs in different age. Yellow, mild case, Red, severe case. **Fig. S4.** Gene marker identify algorithm (LOOCV) to find microorganisms gene marker index distinguishing Severe & Mild cases. A: minimum redundancy–maximum relevance (mRMR) feature selection method and leave-one-out cross-validation (LOOCV) steps. ‘x’ means any genes and ‘a’ means weight of the gene in the formula, it can be plus and minus. B: Find the optimum lowest error rate subset to build a linear discrimination classifier. **Fig. S5.** The rpkm value of all 20 gene markers for the mild and severe case distinguish index model. Yellow, mild case, Red, severe case. The annotation of gene markers were list left together with the weight of each gene in the mRMR selection method. The red box highlight the shared 2 genes in the model build from samples only EV71 positive. **Fig. S6.** EV71 positive samples index model for distinguish severe and mild case. A: leave-one-out cross-validation (LOOCV) to find the optimum subset to build a linear discrimination classifier (18 genes, Training: M:S = 31:35). B: For each individual, an index was calculated to evaluate the risk of severe HFMD disease. The histogram shows the distribution of indices for all individuals. Red means mild case and blue means severe case. C: new samples for test use the index model (Verify: M:S = 10:10). Lowest error rate: 16.67%. Mild case and severe case were distinguished by the index model (p < 0.05). D: Venn chart for the two index model. ev + means the model was built from the samples with all enterovirus positive and ev71 + mean the index model was built from the samples with only enterovirus 71 positive. **Fig. S7. **Intestinal microorganisms difference between EV71 and CV-A groups. A: gene profile between CV-A and EV71 group. B: species profile between CV-A and EV71 group. (Wilcoxon rank-sum test, p < 0.05). C: MLG species enrichment in EV71 group. The size of the circle indicates abundance of the MLG. The color of the circle indicates their taxonomic assignment. Connecting lines represent Spearman correlation coefficient values above 0.6 (grey) and below − 0.6 (blue).

## Data Availability

The datasets used and/or analysed during the current study are available from the corresponding author on reasonable request.

## Abbreviations

HFMD: Hand foot and mouth disease
EV71: Human enterovirus 71
CV-A16: Coxsackievirus A16
CV-A4: Coxsackievirus A4
CV-A10: Coxsackievirus A10
BGI-IRB: Institutional Review Broad
DEGs: Differentially expressed genes
mRMR: Minimum redundancy–maximum relevance
LOOCV: Leave-one-out cross-validation
MLG: Metagenomic linkage group
IGC: Integrated reference genome of the human gut microbiome
KEGG: Kyoto encyclopedia of genes and genomes
FDR: False discovery rate
