# Supplementary material for: Intestinal microbiota has important effect on severity of hand foot and mouth disease in children
Source: BMC Infect Dis. 2021 Oct 13;21:1062. doi: 10.1186/s12879-021-06748-7 (PMC8513321; doi:10.1186/s12879-021-06748-7)
Supplement: Supplementary file 1 — Additional file 1: Fig. S1. Best parameter select for Meta-transcript linkage group cluster. Red represents all 50 species identified; gray represents more than 30, less than or equal to 49; less than 30 is not shown. Fig. S2. Enterovirus phylogenetic tree between mild & severe case using whole genome wide. A: the phylogenetic tree of the enterovirus 71. B: the phylogenetic tree of the coxsackievirus A4. Red color highlight in branch means mild case and black means severe case. The branch name started with “gi” as the reference sequences. Fig. S3. Proportion of DEGs in different age. Yellow, mild case, Red, severe case. Fig. S4. Gene marker identify algorithm (LOOCV) to find microorganisms gene marker index distinguishing Severe & Mild cases. A: minimum redundancy–maximum relevance (mRMR) feature selection method and leave-one-out cross-validation (LOOCV) steps. ‘x’ means any genes and ‘a’ means weight of the gene in the formula, it can be plus and minus. B: Find the optimum lowest error rate subset to build a linear discrimination classifier. Fig. S5. The rpkm value of all 20 gene markers for the mild and severe case distinguish index model. Yellow, mild case, Red, severe case. The annotation of gene markers were list left together with the weight of each gene in the mRMR selection method. The red box highlight the shared 2 genes in the model build from samples only EV71 positive. Fig. S6. EV71 positive samples index model for distinguish severe and mild case. A: leave-one-out cross-validation (LOOCV) to find the optimum subset to build a linear discrimination classifier (18 genes, Training: M:S = 31:35). B: For each individual, an index was calculated to evaluate the risk of severe HFMD disease. The histogram shows the distribution of indices for all individuals. Red means mild case and blue means severe case. C: new samples for test use the index model (Verify: M:S = 10:10). Lowest error rate: 16.67%. Mild case and severe case were distinguished by the index mode [file 12879_2021_6748_MOESM1_ESM.doc]

**Supplementray Materials for**

**Intestinal Microbiota Plays Important Effect on Severity of Hand Foot and Mouth Disease in Children**

## Figures


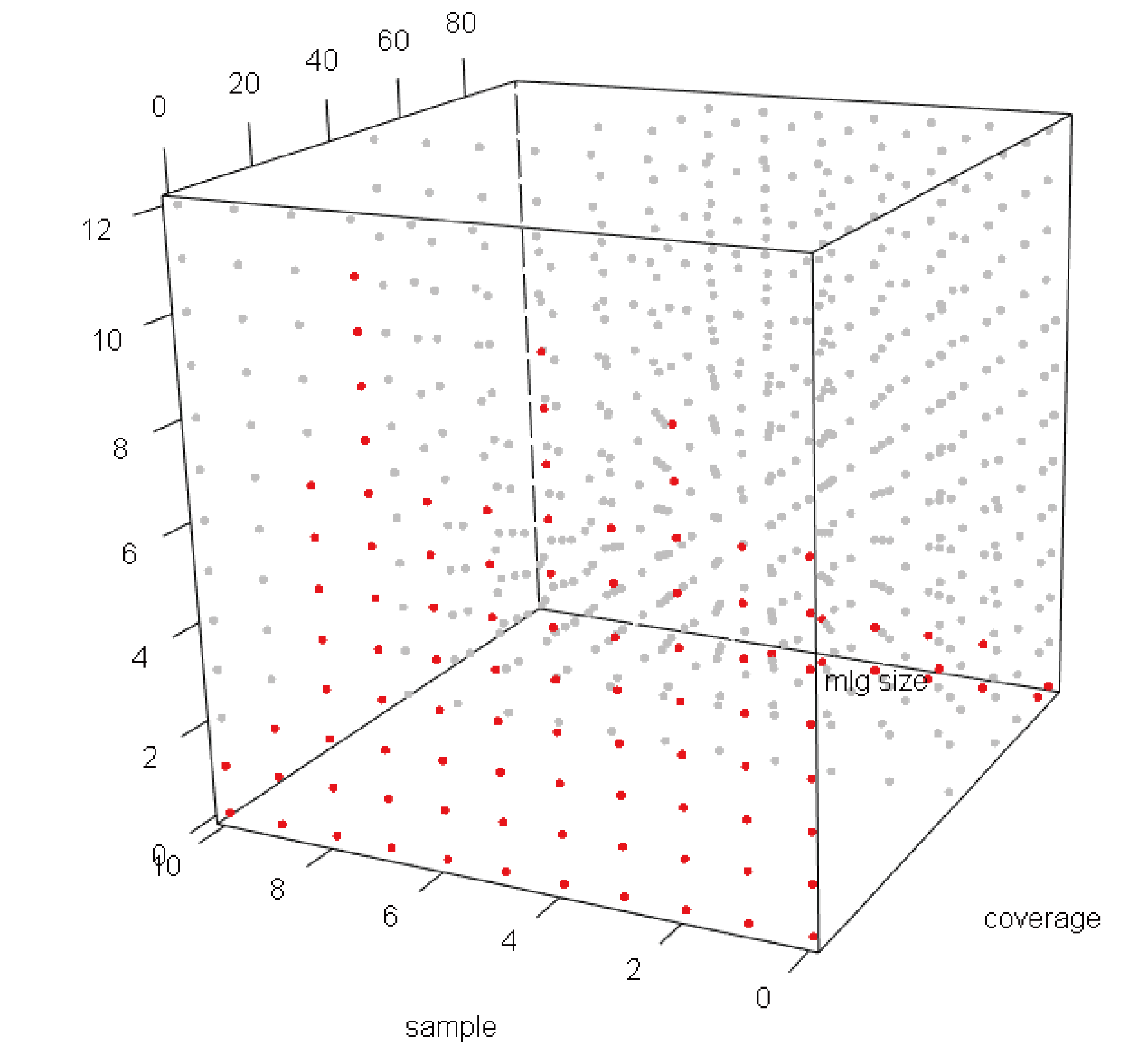


**Supplementary Figure 1. Best parameter select for Meta-transcript linkage group cluster.** Red represents all 50 species identified; gray represents more than 30, less than or equal to 49; less than 30 is not shown.


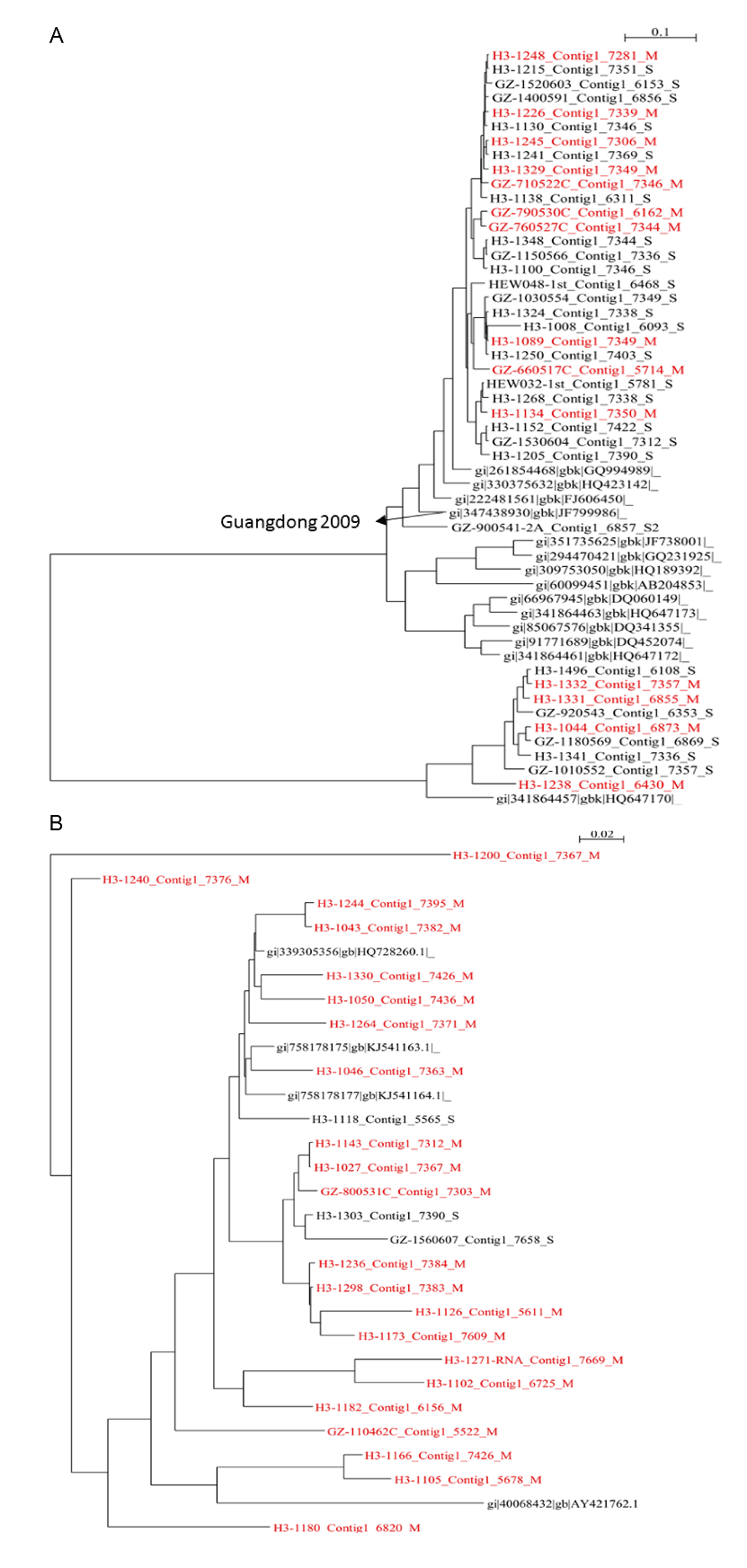


**Supplementary Figure 2. Enterovirus phylogenetic tree between mild & severe case using whole genome wide.** A: the phylogenetic tree of the enterovirus 71. B: the phylogenetic tree of the coxsackievirus A4. Red color highlight in branch means mild case and black means severe case. The branch name started with “gi” as the reference sequences.


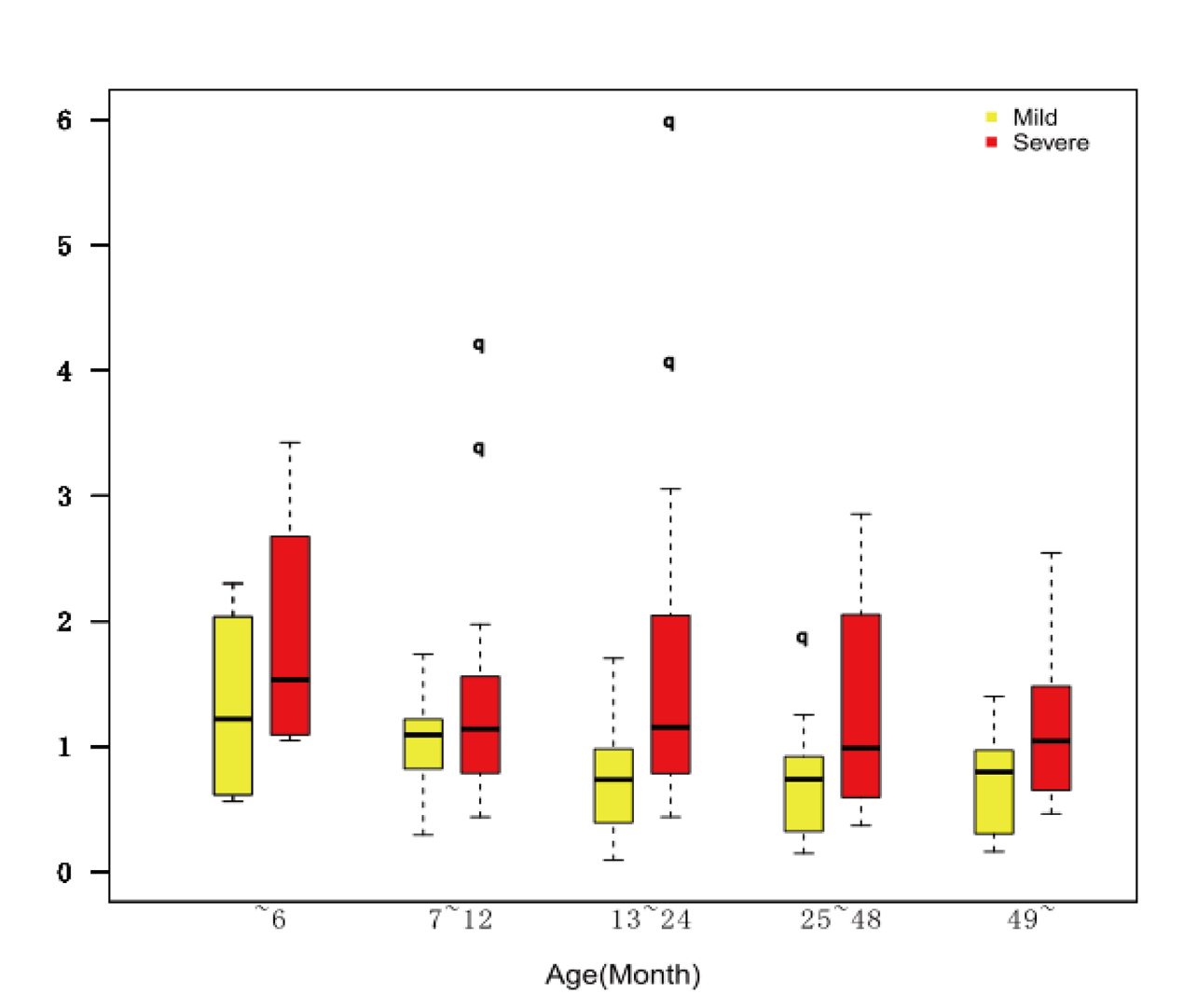


**Supplementary Figure 3. Proportion of DEGs in different age.** Yellow, mild case, Red, severe case.


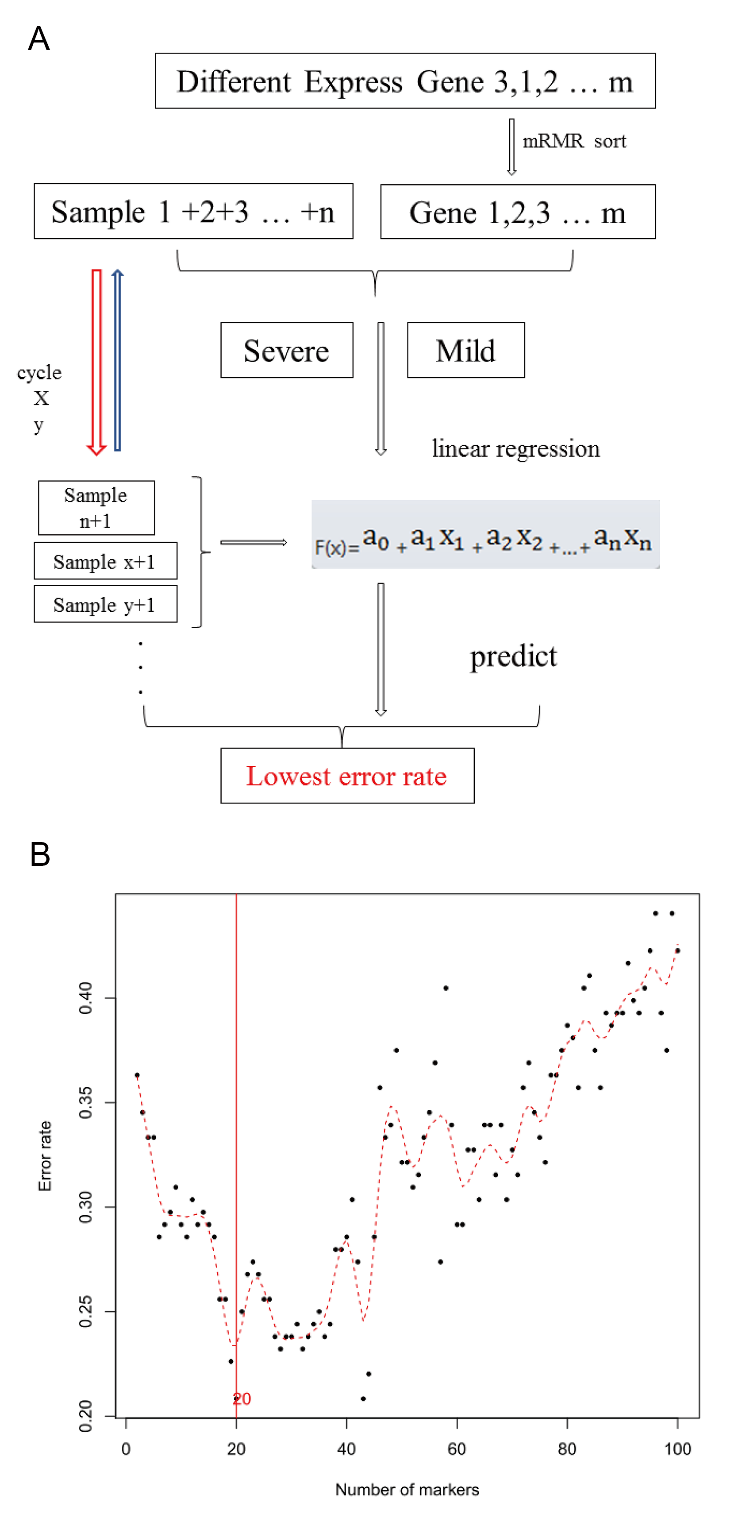


**Supplementary Figure 4. Gene marker identify algorithm (LOOCV) to find microorganisms gene marker index distinguish Severe & Mild cases.** A: minimum redundancy–maximum relevance (mRMR) feature selection method and leave-one-out cross-validation (LOOCV) steps. ‘x’ means any genes and ‘a’ means weight of the gene in the formula, it can be plus and minus. B: Find the optimum lowest error rate subset to build a linear discrimination classifier.


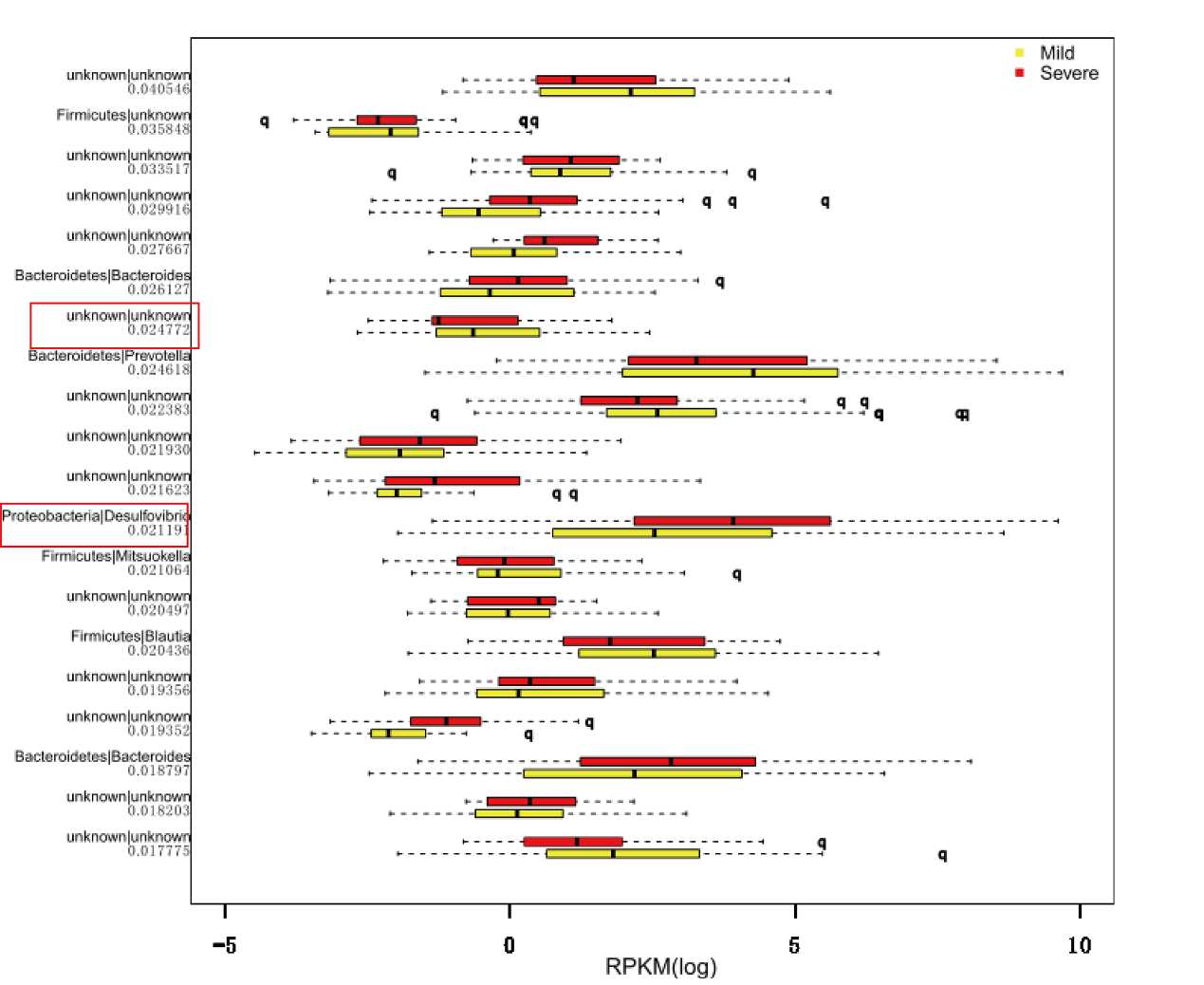


**Supplementary Figure 5. The rpkm value of all 20 gene markers for the mild and severe case distinguish index model.** Yellow, mild case, Red, severe case. The annotation of gene markers were list left together with the weight of each gene in the mRMR selection method. The red box highlight the shared 2 genes in the model build from samples only EV71 positive.


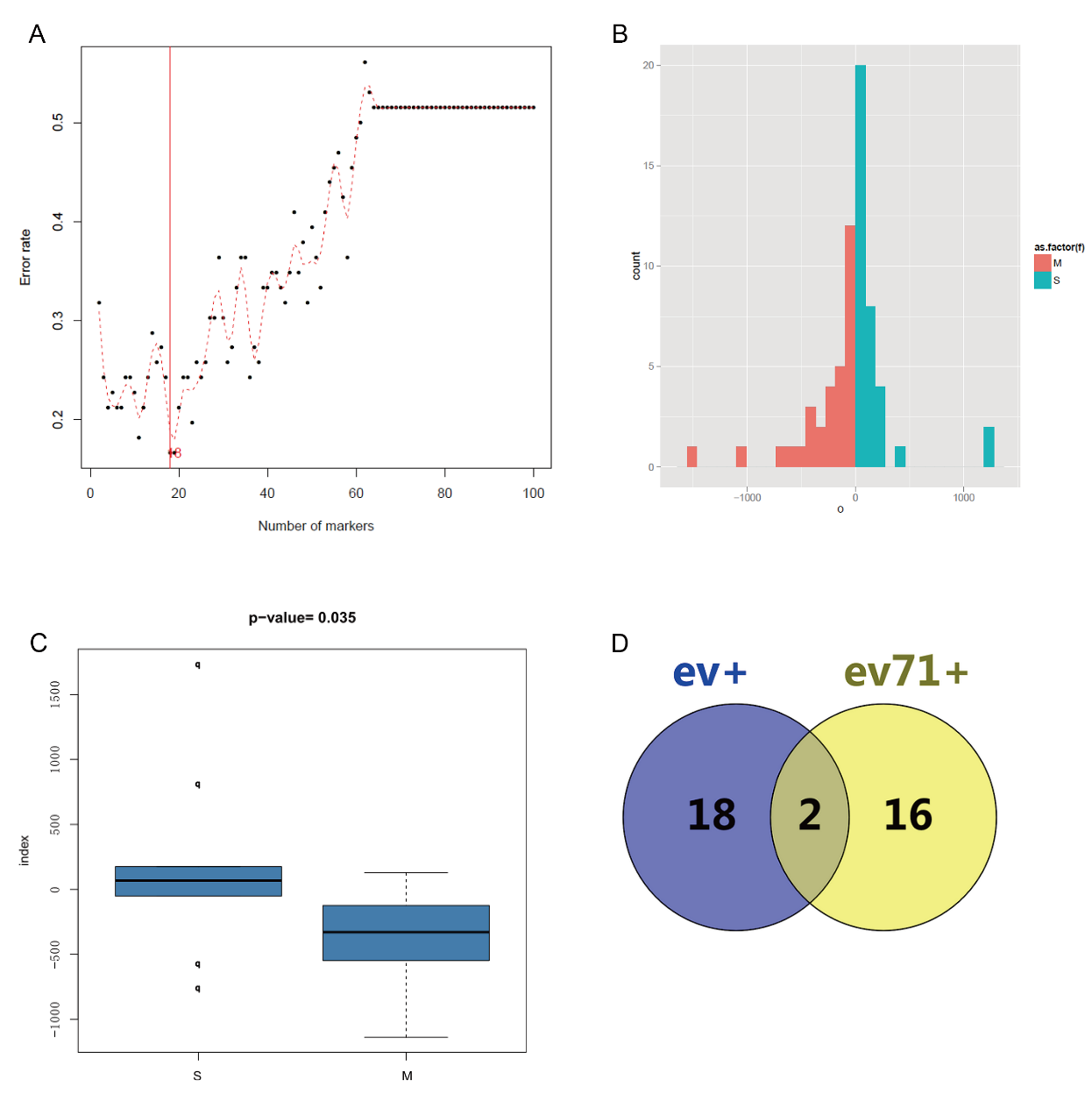


**Supplementary Figure 6. EV71 positive samples index model for distinguish severe and mild case.** A: leave-one-out cross-validation (LOOCV) to find the optimum subset to build a linear discrimination classifier (18 genes, Training: M:S=31:35). B: For each individual, a index was calculated to evaluate the risk of severe HFMD disease. The histogram shows the distribution of indices for all individuals. Red means mild case and blue means severe case. C: new samples for test use the index model (Verify: M:S=10:10). Lowest error rate: 16.67%. Mild case and severe case were distinguished by the index model (p<0.05). D: Venn chart for the two index model. ev+ means the model was build from the samples with all enterovirus positive and ev71+ mean the index model was built from the samples with only enterovirus 71 positive.


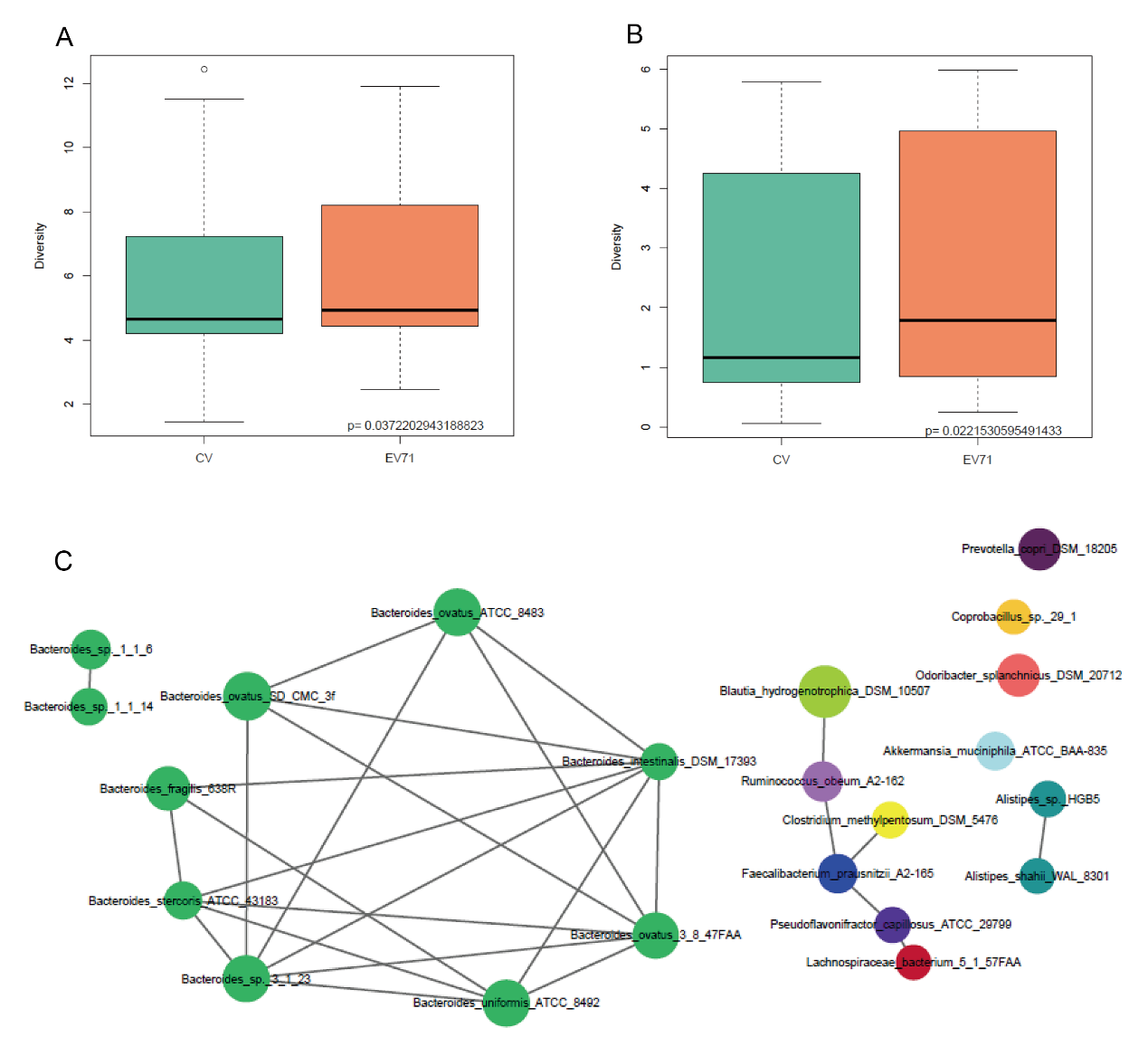


**Supplementary Figure 7. Intestinal microorganisms difference between EV71 and CV-A groups.** A: gene profile between CV-A and EV71 group. B: species profile between CV-A and EV71 group. (Wilcoxon rank-sum test, p<0.05). C: MLG species enrichment in EV71 group. The size of the circle indicates abundance of the MLG. The color of the circle indicates their taxonomic assignment. Connecting lines represent Spearman correlation coefficient values above 0.6 (grey) and below-0.6(blue).
